# Using DFTB to Model Photocatalytic Anatase–Rutile TiO_2_ Nanocrystalline Interfaces and Their Band Alignment

**DOI:** 10.1021/acs.jctc.1c00399

**Published:** 2021-07-07

**Authors:** Verena Kristin Gupta, Bálint Aradi, Kyoung Kweon, Nathan Keilbart, Nir Goldman, Thomas Frauenheim, Jolla Kullgren

**Affiliations:** † Bremen Center for Computational Materials Science, University of Bremen, P.O. Box 330440, D-28334 Bremen, Germany; ‡ Physical and Life Sciences Directorate, Lawrence Livermore National Laboratory, Livermore, California 94550, United States; § Department of Chemical Engineering, University of California, Davis, California 95616, United States; ∥ Computational Science Research Center, No. 10 East Xibeiwang Road, Beijing 100193, China; ⊥ Computational Science and Applied Research Institute, Shenzhen 75120, China; # Department of Chemistry, Structural Chemistry, Angström Laboratory, Uppsala University, Box 538, 752 21 Uppsala, Sweden

## Abstract

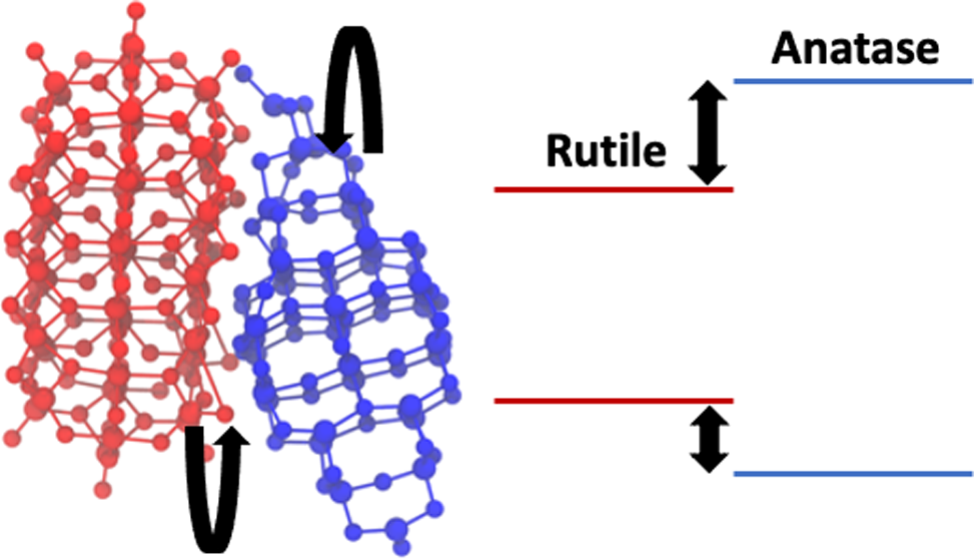

Band alignment effects of anatase and rutile nanocrystals in TiO_2_ powders lead to electron–hole separation, increasing the photocatalytic efficiency of these powders. While size effects and types of possible alignments have been extensively studied, the effect of interface geometries of bonded nanocrystal structures on the alignment is poorly understood. To allow conclusive studies of a vast variety of bonded systems in different orientations, we have developed a new density functional tight-binding parameter set to properly describe quantum confinement in nanocrystals. By applying this set, we found a quantitative influence of the interface structure on the band alignment.

## Introduction

TiO_2_ powders commonly used for photocatalytic applications such as Degussa P25 (DP25) consist of anatase and rutile nanocrystals in different compositions (commonly ∼80% anatase and ∼20% rutile). This composition of the two different TiO_2_ phases is shown to have a great impact on photocatalytic efficiency due to band alignment effects that lead to effective electron–hole pair separation. There have been several theoretical attempts to predict the type of band alignment and thus which crystal acts as a hole or electron trap.^1−4^ Experiments indicate that for the case of DP25, anatase acts as an electron trap, while rutile as a hole trap.^5^ Theoretical works have so far been focused on isolated bulk,^1^ slab,^2^ or nanocrystal^3,4^ systems. While bulk studies predict the trapping of electrons on anatase and the trapping of holes on rutile in accordance with the experiment, nanocrystal studies predict a significant size dependence for the alignment until bulk-like behavior is reached. However, neither of those approaches considers the potential impact of different types of additional degrees of freedom in materials such as geometrical interface arrangements. The role of such interface arrangements was investigated in ref (2) using slab models. However, such models can only give limited insights into the interface effects as the slabs of the two phases must be commensurate, allowing only for a few possible orientations and surface geometries. This makes it impossible to explore the degrees of freedom in the interface formation, which are present under experimental conditions at the microscale.

The vast phase space of possible nanocrystal orientations and sizes thus makes computational studies of anatase–rutile nanocrystal interfaces very challenging, even for very small nanocrystal models where a conclusive study on the role of the interfaces would be unfeasible using density functional theory (DFT)-based calculations alone. Consequently, we have developed a density functional tight-binding (DFTB) parameter set to efficiently simulate TiO_2_ nanocrystals and anatase–rutile interfaces. DFTB, being 2–3 orders of magnitude faster than comparable DFT calculations, provides the possibility of studying a vast number of systems and system sizes. Additionally, with the right choice of parameters, DFTB retains most of the accuracy of DFT and thus yields the possibility of making a closer to one-to-one comparison to experiments. Another benefit of the DFTB approach is its localized basis set that simplifies the computation of non-periodic structures such as nanocrystals.

In the present work, we identify several issues in describing nanocrystals with the previously published DFTB parameter sets for titanium dioxide,^6,7^ e.g., problems in describing the quantum confinement effects correctly and yielding wrong relaxations when undercoordinated species are involved. The latter problem has also been reported in ref (8). To remedy these issues, we have developed a new parameter set, named tio2nano,^9^ that focuses on the correct description of TiO_2_ nanocrystals and interfaces. It is based on the 3ob parameter set.^10^ The electronic parameters of the new Ti species were tailored to ensure the proper description of the quantum confinement effects in TiO_2_ nanocrystals. Instead of the traditional two-center potentials, we have used a many-body force field^11,12^ to represent the repulsive contributions to the total energy, following the same approach as in ref (13). The three-center contributions of the force field allowed us to obtain improved agreement with DFT-optimized geometries compared to the results from two-center repulsive potentials only.

For the band alignment studies, we considered explicit anatase–rutile interface structures. We are not aware of any previous attempts to determine the electronic structure using such models, but similar CeO_2_ nanoparticle models were addressed in ref (14). By applying the newly developed DFTB parameter set on the generated interface structures, we were able to demonstrate the impact of the geometric alignment of the crystals on the band alignment.

## Methods

DFTB calculations were performed with the DFTB+ code.^15^ As described below in detail, the 3ob parameter set^10^ has been extended with element Ti using a density compression radius of 8.5 b and wave compression radii of 5.609, 3.958, and 7.0 b for the s-, p-, and d-orbitals of the Ti atom, respectively. The energy and the chemical hardness (also known as (a.k.a.) the Hubbard *U* value) of the virtual 4p orbital of the Ti atom have been set to 0.206 and −0.08 hartree, respectively.

The energies were created using the Chebyshev interaction model for efficient simulation (ChIMES),^11,12^ a reactive many-body molecular dynamics (MD) force field. ChIMES creates many-body interactions by projecting DFT-computed data (e.g., forces, stress tensors, and energies) onto linear combinations of many-body Chebyshev polynomials.^16^ Briefly, this begins with an *N*-body expansion of the total energy for a system.
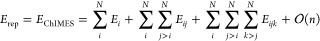
1Here, *E*_*i*_ is the single-atom energy for a given element, *E*_*ij*_ and *E*_*ijk*_ represent the two-body and three-body interaction energies, respectively, *N* is the total number of atoms in the system, and  corresponds to higher-order terms.

Specifically, the two-body interactions are expressed as a linear combination of Chebyshev polynomials of the first kind
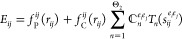
2In this case, *T*_*n*_(*s*_*ij*_) is the Chebyshev polynomial of the first kind of *n*th order, *e*_*i*_ and *e*_*j*_ are the element types of atoms *i* and *j*, and *s*_*ij*_ is a transformation of the interatomic distance *r*_*ij*_ over the Chebyshev interval of [−1,1] using a Morse-like function. In addition, Θ_2_ corresponds to the two-body polynomial order, *f*_C_^*ij*^(*r*_*ij*_) is the cutoff function that ensures the potential and its derivative vary smoothly to zero beyond a specified distance, and *f*_P_^*ij*^(*r*_*ij*_) is a penalty function^13,17^ that helps to prevent sampling of interatomic distances below those seen in the training set (see ref (18) for further details).  is a set of permutationally invariant coefficients of linear combination for a given atom pair type that are determined via a linear least-squares method.

Similarly, three-body interaction energies are expressed as a product of Chebyshev polynomials for the constituent atom pairs of a given triplet, yielding an orthogonal three-body polynomial with  total interactions for a given triplet
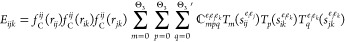
3In this case, Θ_3_ is used to label the three-body polynomial order with a single permutationally invariant coefficient  for each set of triplet atom types. To guarantee that only three-body interactions between *i*, *j*, and *k* are counted toward the sum, only terms for which at least two of the three *m*, *p*, and *q* indices are greater than zero are included in the sum (indicated by the prime in eq 3). In this way, many-body interactions can be included in the repulsive energy (*E*_rep_) by solving a linear least-squares optimization problem where optimal coefficients of the linear combination are determined directly. This avoids reliance on iterative approaches that are required for nonlinear optimization problems (e.g., Levenberg–Marquardt) that are usually more computationally time-consuming and not guaranteed to result in the global minimum. ChIMES models have been extended to include four-body interactions in a similar fashion in reactive MD simulations,^16,19^ though truncation of the ChIMES total energy with the three-body term has proven sufficient for determination of the DFTB repulsive energy. Note that DFTB in its original formulation uses a consistent two-center approximation in both the Hamiltonian matrix elements and the repulsive energy. Our model breaks this consistency by extending only the latter with three-body terms. Extending the Hamiltonian with three-body terms would be more involved, as one would have to deal with the arising pseudopotential-like contributions.^20^ Therefore, while our approach is not a systematic extension, it nevertheless represents a simple and pragmatic way of overcoming some of the deficiencies in the original DFTB formulation.

Our training set for the ChIMES force field was determined from DFT-MD simulations of the amorphous TiO_2_ run at temperatures of 2250 and 300 K using the Vienna Ab initio Simulation Package (VASP) code.^21^ For these calculations, we used the PBEsol functional^22^ with an energy cutoff of 550 eV and Γ-point sampling of the Brillouin zone. PBEsol was chosen due to its improved description of solids and their surfaces. The amorphous phase allowed for improved sampling of a wide range of interatomic distances over the short-time scales of the simulations, which was enhanced by including data from elevated temperatures. Each MD simulation was run on a system of 216 atoms for a total of 5 ps, with configurations taken for *E*_rep_ training every 100 fs (to allow for decoupling between training data), resulting in 50 training configurations taken from each temperature. We also included additional 10 configurations from an MD simulation run at 40 GPa to improve sampling of close interatomic distances. This yielded a total of 110 training configurations.

The training set to determine *E*_rep_ was then computed by subtracting the gradient of the DFTB electronic energy from our DFT reference property, i.e., *F⃗*_TRAIN_ = *F⃗*_DFT_ – *F⃗*_DFTB-elec_. In this work, a spline repulsive fitted to a Ti–Ti dimer was included in the DFTB electronic calculations to ensure that excessively small interatomic distances were not approached during calculations. In addition, O–O distances were poorly sampled in our training set and these repulsive parameters were thus taken from the 3ob-0-1 parameter set and were not a part of our fit. ChIMES parameters were determined through linear least-squares fitting to the resulting ionic forces and the diagonal components of the stress tensor for each of these MD snapshots. We set the ChIMES two-body polynomial order to 12 and the three-body order to 8, similar to previous work,^23^ and solve for optimal coefficients using the least-angle regression (LARS)^24,25^ algorithm with a least absolute shrinkage and selection operator (LASSO)^26^ regularization value of 10^–4^.

For the DFTB calculations, charges were converged with a tolerance of 10^–6^ au and forces with 10^–4^ au. Bulk calculations were performed with an 8 × 8 × 8 Monkhorst–Pack^27^ grid for the primitive unit cell. The rutile (110) surface was constructed as a 4 × 2 surface unit cell with six layers, the (100) surface as a 4 × 2 unit cell with six layers, and the (001) surface as a 4 × 4 unit cell with eight layers. The (001) anatase surface had a 2 × 2 unit cell with eight layers and the (101) surface had an 8 × 4 unit cell with five layers. All rutile structures were computed with a 2 × 2 × 1 *k*-point mesh, the anatase (001) surface with a 4 × 4 × 1 mesh, and the (101) surface with Γ-only calculations. Branching point energy calculations (discussed below) were performed with the primitive unit cell of the anatase and rutile crystals using an 8 × 8 × 8 *k*-point grid. For nanocrystals, we performed Γ-only calculations.

The nanocrystal interfaces were constructed using the JANUS code, which is based on a quick hull algorithm for convex hulls.^28^ This algorithm allows us to rapidly identify convex hulls from a set of points; in this case, the coordinates of atoms from the nanocrystals are in the *xyz* format. The code then combines simplices of the computed convex hull for all normals that form an arccos of 0.99 or larger. The largest facets of each individual nanocrystal are then aligned to each other so that their surface normals are parallel to each other and to the *x*-axis with a user-specified distance between them. The procedure is repeated with the nanocrystals being rotated with respect to each other around the *x*-axis for angles between 0 and 180° in steps of 15°. Additionally, the nanocrystal gets displaced in the *yz*-plane along the perimeter of a circle with a user-specified radius. This displacement occurs with angles between 0 and 360° in steps of 30°. This results in 156 interface geometries, which are stored in an atomic simulation environment (ASE)^29^ trajectory format. To create the interfaces, we used the two smallest relaxed nanocrystals of anatase and rutile. The nanocrystals were aligned with a distance along *x* of 2 Å, and a radius of 2 Å was used for the displacement in the *yz*-plane.

Interface energies were computed as
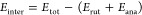
4where *E*_tot_ is the energy of the interface structure, *E*_rut_ is the energy of the isolated rutile nanocrystal contained in the interface structure, and *E*_ana_ accordingly is the energy of the anatase nanocrystal.

For comparative DFT calculations, we used the Vienna Ab initio Simulation Package^21^ (VASP 5.4.4) with the projector augmented wave (PAW) method to describe the cores. For titanium, the 3p electrons were treated as valence electrons. The plane wave cutoff was chosen to be 420 eV and the augmentation cutoff was chosen as 840 eV. Geometry relaxations were performed until energy differences were converged below an error of 10^–3^ eV. The *k*-point meshes were chosen in accordance with the DFTB calculations.

As nanocrystal models, we chose Wulff-type crystal structures for anatase nanocrystals, which expose (101) facets, and quasi Wulff-type crystals for rutile, exposing (110), (100), and (101) facets. These nanocrystal structures were chosen in accordance with ref (3) to allow for direct comparison. Additionally, for the reasons described below, we also considered another set of quasi Wulff-type rutile nanocrystals by cutting particles along the (110) and (101) planes with an even number of (110) layers across the waist of the particle.

## Results and Discussion

### Parameterization and Validation of the New DFTB Parameter Set

**Figure 1:**
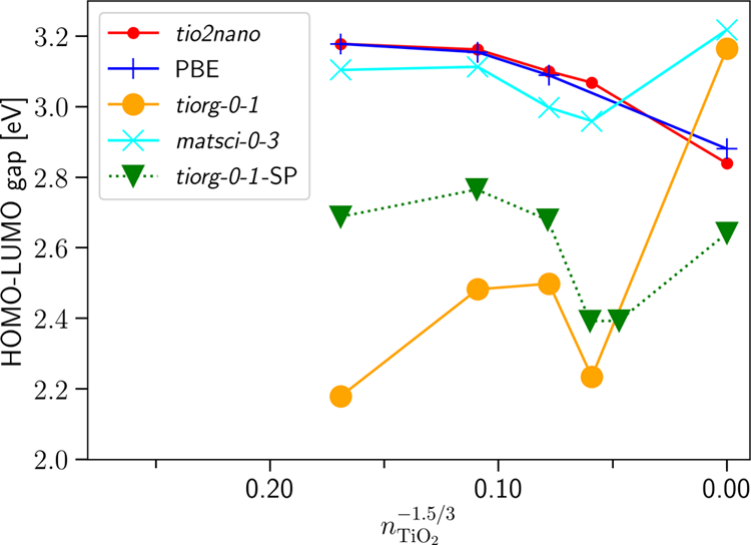
Calculated HOMO–LUMO gaps of anatase nanocrystals using PBE and DFTB with the tiorg-0-1, matsci-0-3, and the newly developed tio2nano set. The PBE results have been shifted to align with the values obtained by the new parameter set for the smallest nanocrystal. The tiorg-SP values were obtained by carrying out DFTB calculations with the tiorg-0-1 set at the PBE geometries (without relaxing with DFTB).

The existing parameter sets for Ti–O interactions, the tiorg-0-1^7^ and matsci-0-3^6^ sets, were evaluated only for bulk structures and common surfaces so far. By applying them to describe anatase Wulff-type nanocrystals, we faced the issue that the highest occupied molecular orbital (HOMO)–lowest unoccupied molecular orbital (LUMO) gap did not change with the particle size as predicted by the theory of quantum confinement^3^ and as observed in analogous Perdew–Burke–Ernzerhof (PBE) calculations. Instead of the expected linear function of *n*^–2/3^ (with *n* being the number of TiO_2_ units), we observed surface states, which fall into the band gap and give rise to HOMO–LUMO gap sizes lower than the bulk value, as shown in Figure 1. To rule out geometrical effects, we also performed single-point calculations for those nanocrystals using their PBE geometries but found a similar wrong behavior with both sets. Efforts to resolve the issue by changing the compression radii of the Ti atom turned out to be unsuccessful. As a next attempt, we tried to describe the Ti–O interaction by extending the 3ob parameter set^10^ with Ti. The electronic parameters of the Ti atom were optimized by fitting on the theoretically expected linear behavior of the band gap with respect to *n*^–2/3^. For this fit, anatase nanocrystals with sizes of *n* = 84, 165, and 286 and the bulk phase of anatase had been used, resulting in the compression radii reported in the Methods section.

**Figure 2:**
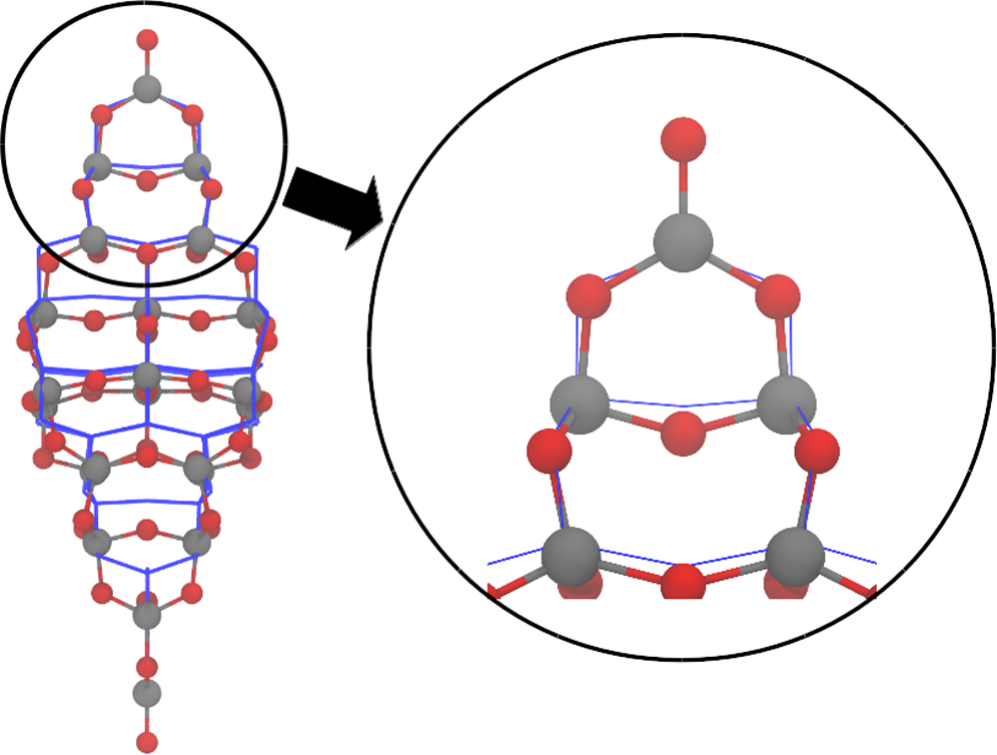
Relaxed geometries of the Ti_35_O_70_ nanocrystal. The DFTB geometry obtained using a two-body repulsive potential for the Ti–O interaction is shown by balls and sticks, while the PBE geometry is represented by blue solid lines. The geometries had been aligned at the top oxygen atom.

After fixing the electronic parameters, the two-center repulsive potentials of the Ti–Ti and the Ti–O interactions had been derived using a symmetrized titanium hexagonal close-packed (hcp) crystal and a symmetrized rutile bulk crystal as references, respectively. The symmetrization ensures that the first neighbor Ti–Ti (in hcp Ti) and Ti–O bonds (in rutile) have identical lengths, allowing for a simple manual fitting procedure. When relaxing the nanocrystals with the obtained repulsive potentials, the resulting geometries exhibited significant deviations in the strongly undercoordinated tip regions as compared to PBE geometries (see Figure 2 for an example). Unfortunately, those geometrical changes lead to the appearance of spurious surface states in the gap. Our attempts to enforce the correct geometry by tuning the two-center repulsive functions were not successful. This is not surprising as the incorrect Ti–O–Ti angle seems to be the main driving force behind the differences in the relaxed geometries. To enforce correct nanocrystal geometries (and with that also the correct electronic structure), we decided to represent the Ti–O and Ti–Ti repulsive functions with the ChIMES force field, which allows the inclusion of three center terms. The interface to ChIMES software had been implemented in a development version of the DFTB+ package. For the Ti–Ti repulsive potential, we kept the two-center one derived from the symmetrized titanium hcp crystal, with ChIMES corrective terms. It is important to note that the performance of the Ti–Ti repulsive potential in describing Ti bulk phases was not evaluated in this work.

The resulting parameterization performs well for anatase and rutile bulk as well as for their surfaces and is able to describe systems for which the previous parameter sets have failed. The bulk lattice parameters given in Table 1 show very good agreement with ab initio counterparts for rutile. For anatase, we observe a slight overestimation of the *a* value, while *c* is underestimated. The calculated surface energies are listed in Table 2. The current parameterization slightly overestimates the surface energies for anatase (001) and rutile (110) compared to the PBE and PBEsol results. The anatase (101) and rutile (001) and (100) surfaces are underestimated compared to the PBEsol results and overestimated compared to PBE. The energetic ordering of the surfaces with respect to each other is reproduced nicely within the tio2nano set. Overall, the current set shows good agreement to ab initio methods.

**Table 1:** Calculated and Experimental Anatase and Rutile Bulk Lattice Parameters (in Å)^A^

	DFTB^a^	PBE^a^	PBEsol^a^	BLYP^b^	exp.^c^
rutile					
*a*	4.629	4.621	4.600	4.679	4.594
*c*	2.980	2.954	2.940	2.985	2.959
anatase					
*a*	3.887	3.792	3.780	3.828	3.784
*c*	9.293	9.640	9.580	9.781	9.515

A Superscripts a, b, and c denote values obtained in this work, ref (30), and ref (31), respectively.

**Table 2:** Calculated Anatase and Rutile Surface Energies in J/m^2^ for Selected Surfaces^A^

	DFTB^a^	PBE^a^	PBEsol^a^	PBE0^b^
anatase				
(001)	1.36	0.95	1.23	1.25*
(101)	0.49	0.45	0.80	0.58
rutile				
(100)	1.07	0.62	1.11	0.83
(110)	0.97	0.37	0.88	0.46
(001)	1.47	1.33	1.79	1.59

A Index a denotes values obtained in this work, while values marked with b were taken from ref (32), except the one indicated by *, which was taken from ref (33).

**Figure 3:**
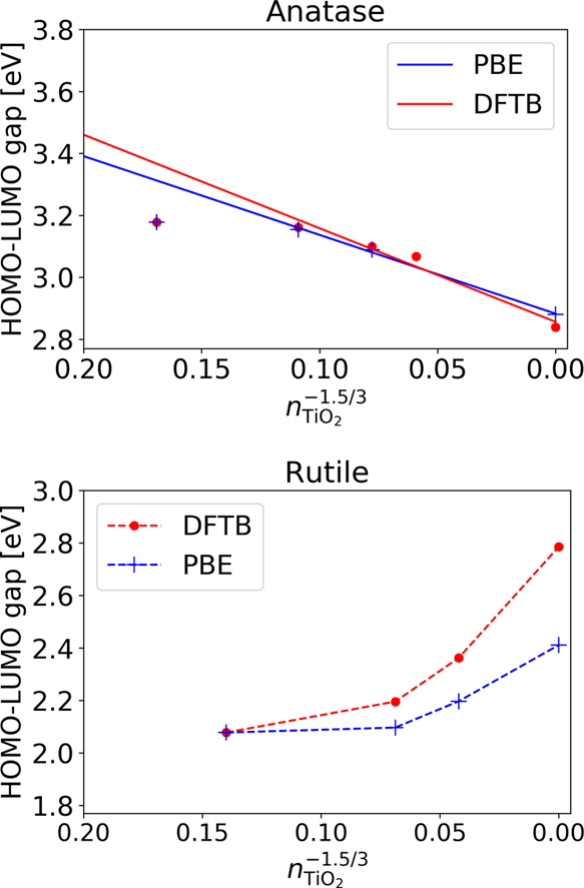
Gap energy behavior over particle size for anatase (top) and rutile (bottom). The DFTB values were obtained with the new parameter set and are displayed in addition to the PBE results. The PBE results were aligned with the DFTB ones to match the value for the smallest nanocrystal structure. Note that for anatase, the smallest crystal size was not included in the linear fit as that structure did not follow the quantum confinement behavior for the gap states. The straight solid lines represent a linear fit, while the dashed lines serve only as guides to the eye.

**Figure 4:**
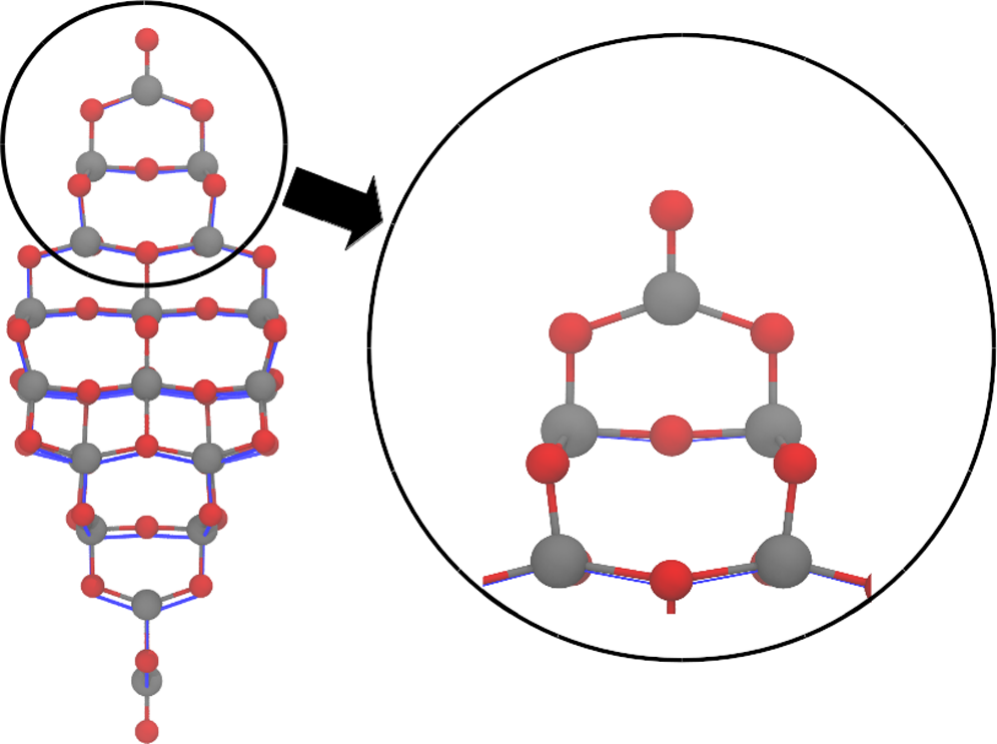
PBE Ti_35_O_70_ relaxed nanocrystal displayed by blue lines aligned at the top oxygen atom to the DFTB relaxation with the new parameter set (shown by sticks and balls). Relaxation at the tip is so close to the PBE counterpart that the blue lines are almost not visible in the representation.

Figure 3 shows the PBE gap values of anatase and rutile in comparison to the ones obtained with the newly developed parameter set. The behavior of both sets is now comparable to ref (3). The smallest anatase nanocrystal considered shows with the ChIMES-based repulsive function a relaxation that is very close to the one obtained with the PBE calculation (see Figure 4). Overall, the new parameters reasonably describe the various forms and phases of titania. The band alignments between the phases are described in the following section.

### Band Alignment

The primary aim of the current paper is to establish a computationally feasible approach that allows us to study band alignment across a TiO_2_ anatase–rutile nanoparticle interface and to determine the role of the interface in this alignment. The approach is based on the customized DFTB parametrization described above. The quality of the new method is assessed by comparison to calculations at the DFT-PBE level of theory.

First, we present the bulk band alignment between anatase and rutile using the branching point energy approach as it was done for TiO_2_ in ref (1) previously and compare the PBE and DFTB values. Afterward, following ref (3), the level alignment deduced from nonbonded nanoparticle structures is discussed. We compare previous results to our DFT-PBE calculations as well as to results obtained with DFTB. Finally, we investigate the influence of the interface on the band alignments by DFTB calculations on bonded nanoparticles.

#### Bulk Band Alignment

**Figure 5:**
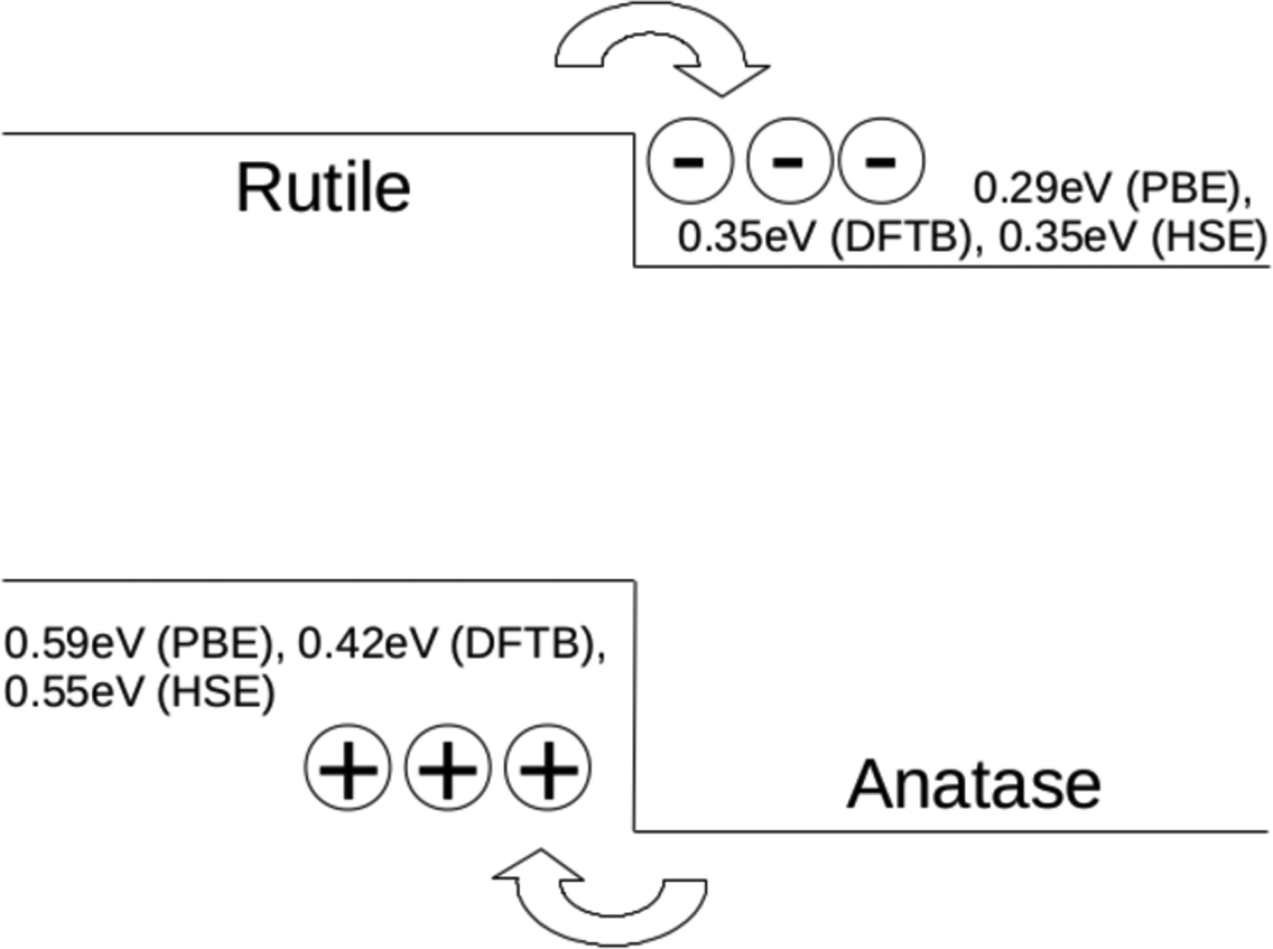
Anatase and rutile band alignments computed by the branching point technique with DFTB (tio2nano), PBE, and HSE06. The HSE06 values were taken from ref (1).

First, we consider the band alignment between the bulk phases using the branching point technique. The branching point energy *E*_BP_, or the charge neutrality level, can be computed from the average of the mid-level states using the relation
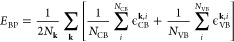
5Index **k** runs over the *N*_**k**_*k*-points being considered. In every *k*-point, *N*_VB_ valence band (VB) and *N*_CB_ conduction band (CB) states with respective eigenvalues ϵ_CB_^**k**,*i*^ and ϵ_VB_^**k**,*i*^ are averaged. We chose *N*_VB_ = 1 and *N*_CB_ = 1 independently of possible degeneracies of the bands, as the alignment is known to depend only weakly on the degeneracy and on the number of included bands.^1^Figure 5 compares our results for *E*_BP_ to those in ref (1). The results obtained with the tio2nano set compare well to the calculated PBE values as well as to the HSE06 values in ref (1). All levels of theory predict that rutile and anatase act as hole and electron traps, respectively.

#### Band Alignment in Nonbonded Nanocrystal Systems

Following ref (3) we performed DFT-PBE and DFTB calculations for anatase and rutile nanocrystals of different sizes. We plotted the HOMO and LUMO levels as well as their gap values as a function of the number of TiO_2_ formula units in the structure raised to the factor −α/3. The parameter α was set to 1.5 as this yielded a better fit to our data than a value of 1.35 used in ref (3). Note that we did not include the smallest anatase crystal in the linear fit, as we found it to not follow the particle in the box model (neither for PBE nor for DFTB). The PBE and DFTB results for anatase are in good agreement with the PBEx data of ref (3). By extrapolating the DFTB nanocrystal results to infinite particle sizes using a linear fit with respect to *n*^–1.5/3^, we obtained a band gap of 3.02 eV as compared to the bulk value of 2.84 eV. A similar overestimation can be seen in the extrapolation of the PBE data, which yields a gap of 2.25 eV as compared to a PBE bulk value of 2.09 eV.

**Figure 6:**
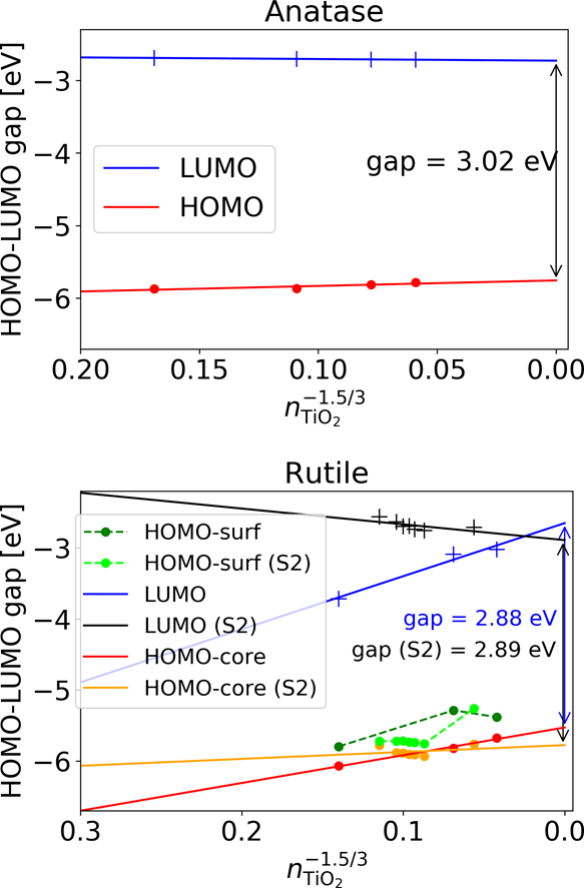
HOMO and LUMO levels for anatase (top) and rutile (bottom) calculated with DFTB. For rutile, both the highest occupied core states (HOMO-core) and the occupied surfaces states (HOMO-surf) are shown. The corresponding levels for the second rutile crystal set are indicated with (S2). The dashed connection lines serve only as guides to the eye. The indicated gap sizes of the bulk phases are obtained from the linear fits of the respective HOMO and LUMO states.

**Figure 7:**
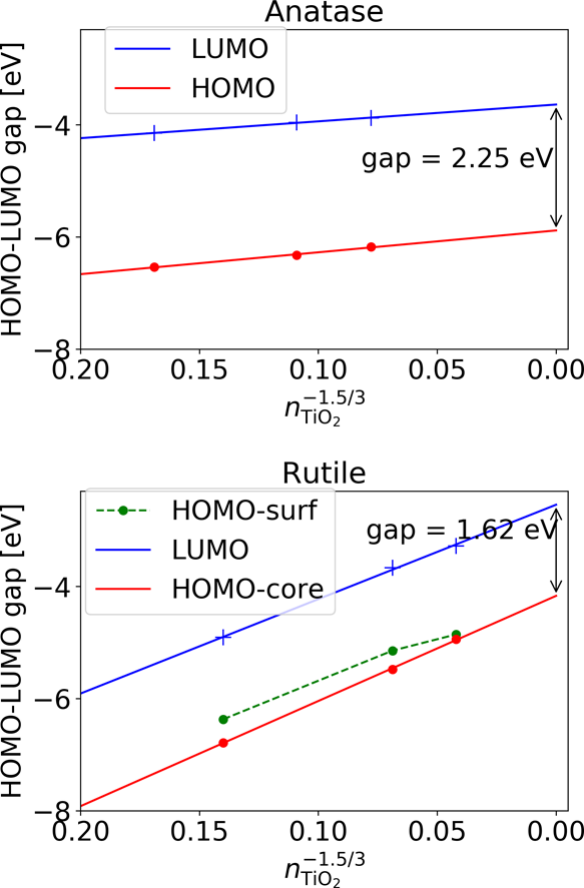
HOMO and LUMO levels for anatase (top) and rutile (bottom) calculated with PBE. For rutile, both the highest occupied core states (HOMO-core) and the occupied surfaces states (HOMO-surf) are shown. The dashed connection lines serve only as guides to the eye. The indicated gap sizes of the bulk phases are obtained from the linear fits of the respective HOMO and LUMO states.

In the case of rutile nanocrystals, extrapolation of the band gap is complicated by the presence of surface states. This leads to the nonlinearity of the band gap caused by the nonlinear behavior of the VB edge. As shown in Figure 6, although the highest lying occupied core states clearly follow the confinement linearly, an occupied surface state with a nonlinear behavior appears in the gap. This leads to an initial narrowing of the gap with the particle size before reaching the point where the surface and core states cross and after which the gap follows a linear behavior again. Similar behavior can be found in the PBE results, as shown in Figure 7. The observed behavior of the surface states indicates that the simple textbook particle in a box model can be applied only to core states but not to surface states.

Comparing the extrapolated value of the band gap for rutile, excluding the surface states, from the nanocrystals with the calculated band gap of the bulk, we find that the extrapolated value slightly overestimates the bulk one with DFTB (2.88 vs 2.76 eV) and underestimates it with PBE (1.62 vs 1.79 eV). We also note that the rutile data indicate an inverse quantum confinement trend even when excluding the surface states, in disagreement with the PBEx data presented in ref (3). We are not entirely sure what the origin of the discrepancy is. We note, however, that both rutile slabs^34^ and nanowires^35^ exclusively exposing (110) surfaces show an even–odd behavior in the valence and conduction band edges with the number of layers. For odd numbers, both display an inverse quantum confinement behavior, while for even numbers, they display regular quantum confinement behavior. The effect was found to be more pronounced for the conduction band states.

**Figure 8:**
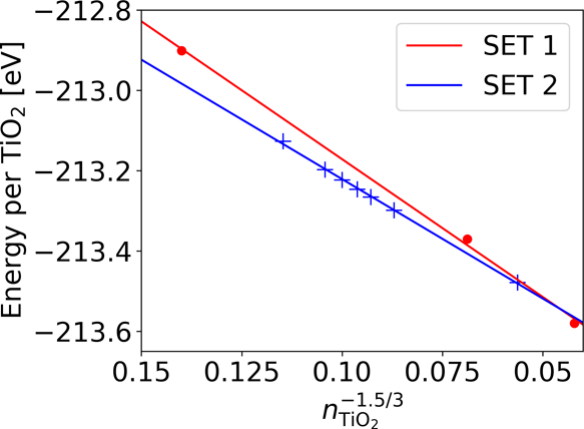
Comparison of the stability of rutile nanocrystals in the original set from ref (3) (SET 1) and the new set (SET 2) as a function of size. Energies are given per TiO_2_ formula units.

Therefore, in addition, we also considered another set of quasi Wulff-type rutile nanocrystals by cutting particles along the (110) and (101) planes with an even number of (110) layers across the waist of the particle. In the following, we refer to this new set as SET 2. These nanocrystals contain 76, 92, 100, 108, 116, 132, and 316 TiO_2_ formula units. To make the crystals stoichiometric, we removed a number of Ti atoms at the (101) facets as opposed to adding dangling oxygen ions as was done in ref (3). The particles of this set are found to be more stable compared to those presented in ref (3) (see Figure 8). We also tried building larger particles with an even number of (110) layers, but these particles became metallic and also significantly less stable. Using the stable and nonmetallic particles of the new set, we obtain an extrapolated value (excluding surface states) for a band gap of 2.89 eV (see Figure 6). We note that the new set displays the expected quantum confinement trend with a decreasing HOMO-core to LUMO gap with increasing crystal size. While the HOMO-core states of the rutile nanocrystals of the new set are located in the same energy region as those of the original set, the LUMO states are located at higher energies. This behavior is therefore consistent with the even–odd behavior with respect to the number of (110) layers for the VB and CB positions for slabs and nanowires in refs (34) and (35), respectively.

Given that particles of comparable stability may show contrasting behavior in terms of their HOMO and LUMO positions, suggesting that we need to be careful in establishing scaling relations—explicit simulations are clearly required for small rutile nanocrystals.

Using the HOMO and LUMO level positions obtained above, band level alignments in combined nanocrystals, consisting of a rutile nanocrystal and an anatase nanocrystal, can be predicted. However, note that such predictions would not take the effect of the actual anatase–rutile interfaces into account, which can have a significant impact (see the presentation in the Band Alignment in Bonded Nanocrystals section). We also note that much of such size dependence in the alignment is driven by the large variation in the HOMO and LUMO positions in the rutile nanocrystals. The results also depend on the type of rutile nanocrystals we use. Nevertheless, combining an anatase nanocrystal with rutile nanocrystals of the type presented in ref (3), one obtains a so-called type II rutile alignment (using the nomenclature of ref (3)) for small rutile nanocrystals, where rutile acts as an electron trap and anatase as a hole trap. Increasing the size of the rutile nanocrystal results in a transition to a so-called type I alignment, where both the HOMO and LUMO states are located within the rutile nanocrystal. Finally, for even larger rutile crystal sizes, one obtains a so-called type II anatase alignment (as also predicted by the branching point calculation with the bulk structures), where rutile acts as a hole and anatase as an electron trap. The same quantitative behavior was derived for PBEx in ref (3).

On the other hand, by combining an anatase nanocrystal with a rutile nanocrystal from the new set (S2), one can obtain a number of different alignments. Most notably, using the smallest rutile nanocrystal of the new set leads to an alignment where both the HOMO and LUMO states are located within the anatase nanocrystal.

With the aforementioned difficulties in establishing robust trends in the HOMO and LUMO levels in rutile nanocrystals, we conclude that we are unable to make a robust prediction in the HOMO–LUMO level alignment between anatase and rutile nanocrystals, at least for the size range covered by our simulations, ∼1–4.5 nm, without resorting to explicit simulations.

#### Band Alignment in Bonded Nanocrystals

**Figure 9:**
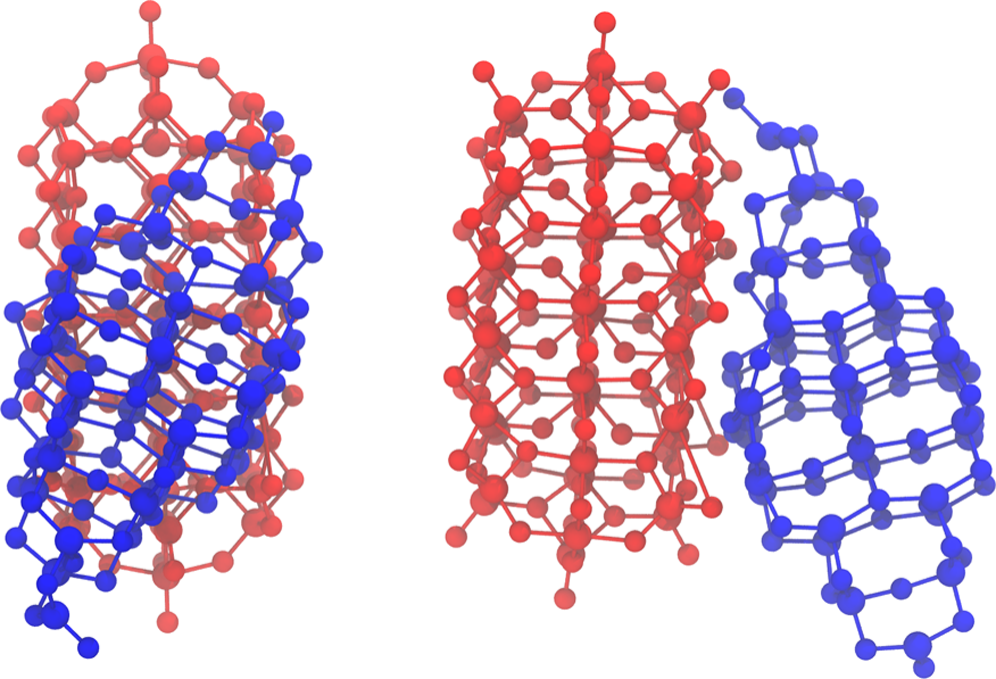
Typical alignment of the most stable rutile–anatase nanocrystal interface structures. The red and blue structures represent the rutile and anatase nanocrystals, respectively.

Exploiting the efficiency of the DFTB method, we have investigated the influence of the interface on the gap alignment by simulating explicit interface structures between different orientations of the smallest anatase and rutile nanocrystals. In the most stable structures, we have obtained nanocrystals with a rutile (110) and anatase (101) interface, and the nanocrystals tend to align with an angle of around 30° with respect to each other along the rutile [001] and anatase [010] directions. This alignment, as shown in Figure 9, maximizes the interaction area and the bond formation between the two structures, minimizing also the interface energy that was computed according to eq 4.

**Figure 10:**
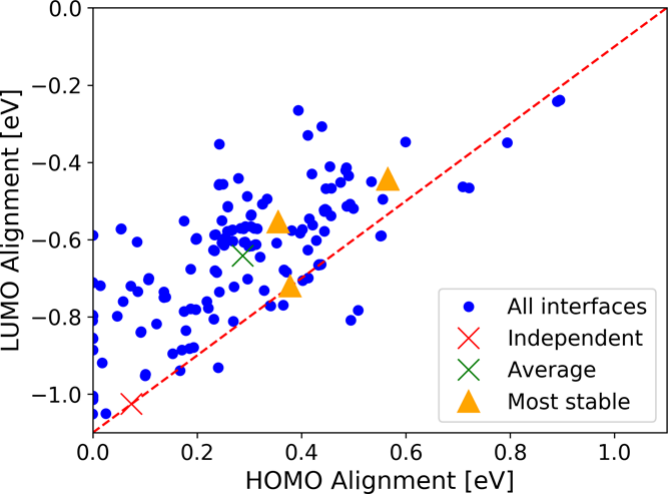
HOMO and LUMO alignment of TiO_2_ nanoparticles composed of rutile and anatase nanocrystals. The alignment is positive if the respective edge state in rutile is higher than in anatase. Blue dots indicate values obtained from bonded nanocrystals, green crosses indicate their average, and the red cross indicates the value obtained from the noninteracting nanocrystals. The five most stable structures are marked with yellow triangles, but since two pairs have very similar alignment values, only three triangles can be seen. The dashed diagonal line serves as a guide to the eye and moving parallel to this line corresponds to alignments with constant gap sizes for both nanocrystals. In the line shown, the values for these gaps are 2.08 eV for rutile and 3.18 eV for anatase, corresponding to the HOMO–LUMO gaps of the isolated particles.

The prediction using independent nanocrystals (neglecting the interface effects) suggests a type I alignment with mismatches of 0.07 eV for the HOMO and 1.03 eV for the LUMO. This suggests complete domination of the gap by rutile states, due to the narrowing of its gap by surface states. The gap alignments for all 156 investigated bonded arrangements, their average, and the value obtained from the noninteracting nanocrystals are displayed in Figure 10. The five configurations with the lowest energy have also been marked in the figure. It is obvious that although the prediction using noninteracting nanocrystals delivers the correct alignment type, the magnitude in the offset is far off from both the average interface offset and the one obtained from the most stable interface structures. This suggests that interface effects have a significant influence on the level alignment and must be accounted for explicitly. One observes a slight correlation of the LUMO/HOMO alignments along with the indicated diagonal line, which would correspond to rigid shifts of the band edges without any change in the band gap. This, therefore, represents situations where bands in both phases are shifted due to an interface dipole that varies in magnitude depending on the relative orientation of the nanocrystals, but where no new interface states are being created.

**Figure 11:**
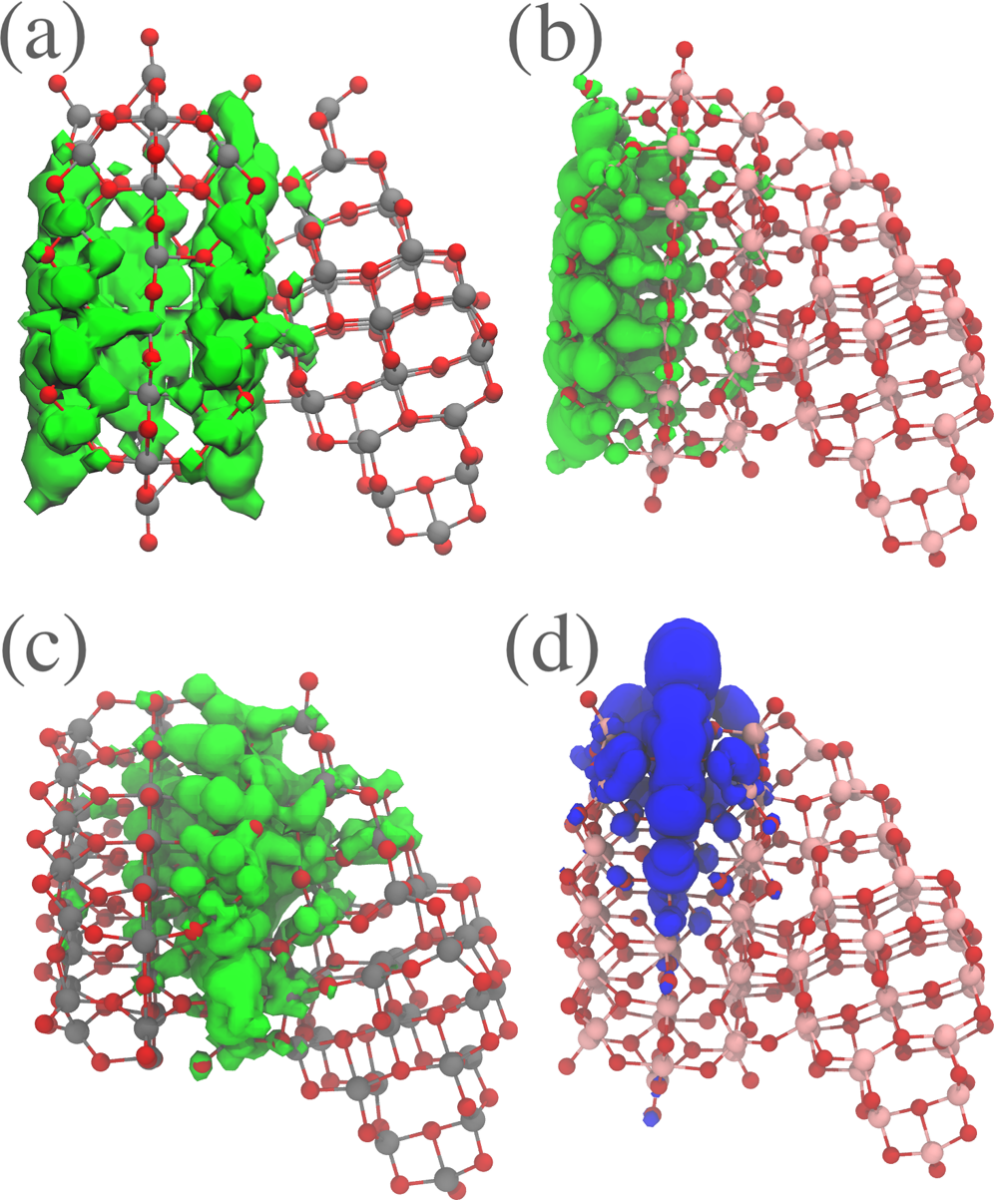
Typical LUMO and HOMO wave functions in the anatase–rutile nanocrystals. (a) The least common LUMO wave function type that is similar to the LUMO of an isolated rutile nanocrystal, (b) the most common interface wave function, where the wave function is not present in the interface region, (c) the second most common interface type, where the LUMO wave function is located at the interface, and (d) the HOMO wave function, which is similar in all interfaces and corresponds to the HOMO surface state of the isolated rutile nanoparticles.

Some typical LUMO and HOMO wave functions of the interfaces are displayed in Figure 11. We found three different types of LUMO wave functions and one HOMO wave function. The HOMO wave function is identical to the rutile HOMO surface state. The most common LUMO wave function is located exclusively in the rutile nanoparticle but differs considerably from the LUMO wave function of the isolated rutile nanocrystal. The second most common is located directly at the interface, while the least common one, which only occurs in four of the investigated cases, resembles the original rutile LUMO wave function. The interface in each of the five most stable structures is of the first type, which is also the most common among all investigated nanoparticles. This underlines the necessity to model nanocrystal interfaces explicitly, as the LUMO edge states might differ considerably from those obtained from the independent particle model. This aspect would be important to consider, not least when modeling catalytic reactions at the surface of nanocrystals.

## Conclusions

We have developed a DFTB parameter set using the ChIMES force field with three-body terms as repulsive potentials especially designed for rutile and anatase nanocrystals. We found that the three-body term was crucial to predict the correct relaxation around the low coordinated atoms at the apex of anatase nanocrystals.

We used the new DFTB parametrization, the tio2nano set, to investigate different anatase–rutile band alignment models to predict effective charge carrier separation in mixed anatase–rutile systems. While the bulk band alignment model predicts a type II anatase alignment where anatase acts as an electron trap and rutile as a hole trap in accordance with the literature,^1,2,5^ the nanocrystal model indicates the strong dependence of the band alignment type on the crystal size in accordance to the predictions in ref (3). The detailed shape of small rutile nanocrystals also plays a crucial role. We showed that two types of rutile nanocrystals that predominantly expose the (110) facets and have similar stability give rise to rather different behaviors in terms of band alignment. This fact also makes it difficult to establish robust rules of thumb when it comes to predicting band alignment in a rutile/anatase nanocrystal mixture and underlines the necessity for explicit simulations of those systems.

Using the efficiency of the DFTB method, we also investigated the effect of the anatase/rutile nanocrystal interface on the band alignment. We optimized the geometry of 156 anatase/rutile nanocrystal pairs where the mutual orientations were systematically varied. While all interface models show the same type I alignment, the magnitude of the band offset varies almost 1 eV with different orientations. This observation suggests that it is important to consider interface effects on the band alignment and underpins the importance of being able to extend the reach of electronic structure simulations beyond the realms spanned by regular DFT.
